# Fertility preservation for transgender individuals

**DOI:** 10.1530/RAF-21-0090

**Published:** 2022-04-22

**Authors:** James Barrett

**Affiliations:** 1 Charing Cross Gender Identity Clinic, Tavistock and Portman NHS Trust, London, UK

## Abstract

Transgender people are just as able to be good parents as anyone else. The treatment involved in addressing their gender dysphoria usually removes their natural fertility and if they want to preserve their fertility, they will need gamete storage. The technology needed to provide gamete storage for transfolk is not any different from anyone else but the clinic setup, human interactions and the approach used need to be sensitive and require everyone in the clinic to understand the issues and behave accordingly if high-quality service is to be provided.

Transpeople are individuals who are not content to live in the gender role that follows from the sex they were assigned on their birth certificate. Instead, they make a change to a different social gender role – usually, the one not assigned on their birth certificate but sometimes, more rarely, to a gender-neutral role. These individuals attract an International Classification of Diseases version 11 diagnosis of gender incongruence (Barrett 2007, Richards & Barrett 2020).

Being trans is not a mental illness or disorder – gender incongruence is ‘a condition related to sexual health’ according to the International Classification of Diseases version 11. There is no connection to sexual orientation – one is who you go to bed *with*, the other who you go to bed *as*.

Nearly all transpeople who have made a change of social gender role seek endocrine treatment. Transwomen take estradiol to elicit an estrogen level in the female follicular range and very often a gonadotrophin-releasing hormone analog to suppress testicular testosterone levels into the normal female range. Transmen take testosterone to elicit a testosterone level in the normal male range, which generally serves to suppress ovarian function. Non-binary people generally seek a hormonal profile somewhere between these two states.

Nearly all transmen undergo a bilateral mastectomy and male chest reconstruction but only about a third seek the complicated, multi-stage surgery needed to create a penis (phalloplasty) which often incorporates a hysterectomy and bilateral salpingo-oophorectomy.

About two-thirds of transwomen seek genital reconstructive surgery – which involves the creation of a vulva and clitoris and very often a neovagina as well. Many self-fund augmentation mammoplasties or facial feminization surgery as neither procedure is currently NHS funded.

The mean age at presentation to a gender identity clinic has fallen over the years and is now such that, for the majority of patients, fertility preservation is an issue. A growing proportion is so young as not to have thought seriously about family formation, always having felt that at their age it is ‘in the future’, but suddenly finding themselves having to think about it immediately, as their natural fertility is threatened by hormone treatment. Impatience to start hormone treatment, with later regret about lost fertility or the need to interrupt treatment to preserve fertility, is a fairly common problem.

Most transpeople seeking to preserve their fertility want to do so before commencing hormone treatment. A few suspend already well-established hormone treatment to preserve their fertility, sometimes having changed their minds late in the day or having been earlier wrongly advised that NHS funding was not available. A very small number suspend completed hormone treatment to impregnate a partner or to themselves become pregnant.

Getting it right for transpeople starts long before the patient actually arrives in your clinic. Has the prior communication used the appropriate pronoun and name? (The disparate NHS records do not all automatically update to reflect this.) Do not send a transman pre-appointment information leaflet that begins ‘when a woman has her eggs collected she...’ but instead consider an across-the-board mild and completely grammatically sound conversion so instead it reads ‘when people have their eggs collected they...’ This re-wording works for every patient having eggs collected, transman and woman alike. Note also that the Human Fertilisation and Embryology Authority provides forms, especially for use by transpeople, that you must use (Information for trans and non-binary people seeking fertility treatment, HFEA).

The rate of autistic spectrum conditions is very much higher in the trans population than in the general population (Warrier *et al*. 2020). Consequently, it might be advisable to make sure that any literature sent to transpeople works well for someone who is autistic by being direct, clear and unambiguous. Information written in this way tends to work pretty well for those who are not autistic, too. In a related vein, it would be sensible to have within your suite of informative videos and webinars some specifically tailored to transpeople (gay and lesbian people too, come to that).

Patients need to be greeted, seated and treated as their authentic selves, in their gender role. Make sure that every member of staff gets it right, the first time, starting with the all-important first contact receptionist all the way through to every orderly and assistant. Accept that this might mean additional training for all; be prepared to be firm with those who express objections and subsequently repeatedly ‘forget’.

Do not allocate patients to a clinic on the basis of the sorts of treatment they are getting but rather on the basis of who they are. Transwomen need to be seen in a clinic that deals with women. The patients in that clinic are having a wide variety of medical consultations and the transwoman is a woman who happens to be consulting about semen storage. Likewise, the transman needs to be seen in a clinic with other men because he is a man, a man who is considering egg freezing. It should go without saying that transmen use the toilet for men and transwomen that for women. There will need to be sanitary towel facilities in both as a result.

Because most transpeople present for fertility preservation before hormone treatment has commenced they do not have any of the physiological feminization or masculinization that later hormone treatment will cause; consequently, they may look androgynous. Be aware that although their appearance might be ambiguous, their sense of themselves is absolutely not. They are as certain of their own identity as any other man or woman in the waiting room, are acutely and painfully aware that their external appearance does not match that certainty and are desperate to get fertility preservation done and dusted so that they can commence hormone treatment and thereby start to look more like themselves. They will be very thankful if, once inside the door of the fertility services, they are treated as who they are, not what they unhappily currently look like.

Facilities for semen production used in the past were designed for men – and heterosexual men to boot. That is no longer the case as there are now women (transwomen) needing to use those same facilities. Ask the women in your department whether the facilities currently used to collect semen samples are a place where they would be comfortable, as a woman, masturbating to orgasm. If they are not, you need to change them until they are. Note also that while most transwomen can produce a semen sample by masturbation, a significant proportion, particularly younger patients, simply cannot do this in any setting, including the privacy of their own home. These patients may need surgical sperm retrieval or electroejaculation, as used for peripubertal boys with childhood malignancies.

Avoid genital exposure or examination unless it is absolutely necessary and there is no alternative. Many transpeople cannot bear to look at their own genitals, let alone let anyone else do so. Always use abdominal scans if it is in any way possible and if the genital examination is unavoidable, consider sedation or anesthesia. If the patient is conscious, bear in mind that many transmen would much prefer an ultrasound probe placed rectally rather than vaginally. Some would rather prefer a man wielding that alarming-looking probe, some a woman. As ever, the golden rule is to ask, to offer a choice if at all possible and then to go with that choice.

In a related vein, for transmen, the area between the shoulder sockets and above the xiphisternum is ‘the chest’ whether or not there are what that look to you like breasts on it. What looks like ordinary breasts to you are unwanted by him and feel alien and inappropriate. Use the term he feels comfortable with, not one that reminds him of his unwanted problems.

Notably, fertility preservation in the context of gender incongruence is funded throughout Scotland and Wales and by virtually all English Clinical Commissioning Groups (CCG). In April 2019, NHS England advised Clinical Commissioning Groups that ‘all patient groups whose medical treatment may compromise fertility should be in the contemplation of a CCG when its clinical commissioning policy for fertility preservation is being developed or is under review. Given the legal duties identified above, CCGs must not determine which patient groups might be offered fertility preservation services on a basis that discriminates against those patients because of a protected characteristic, including gender re-assignment’ (Formation of clinical commissioning policies for fertility preservation: Guidance for Clinical Commissioning Groups, NHS).

Finally, after some time, it is likely exactly the same patients will return in connection with assisted fertility (looking very much less androgynous and decidedly more cheerful as a result of this pleasing change). Because their gametes were preserved, they are to be used for inter-partner donation and the patients must be advised that because they are acting as a donor, the laws stipulated by the HFEA will oblige them to undergo the relevant screening processes.

## Declaration of interest

The author declares that there is no conflict of interest that could be perceived as prejudicing the impartiality of this commentary.

## Funding

This study did not receive any specific grant from any funding agency in the public, commercial or not-for-profit sector.
